# Folding Circular Permutants of IL-1β: Route Selection Driven by Functional Frustration

**DOI:** 10.1371/journal.pone.0038512

**Published:** 2012-06-05

**Authors:** Dominique T. Capraro, Shachi Gosavi, Melinda Roy, José N. Onuchic, Patricia A. Jennings

**Affiliations:** 1 Center for Theoretical Biological Physics, Rice University, Houston, Texas, United States of America; 2 National Centre for Biological Sciences, Tata Institute of Fundamental Research, Bangalore, India; 3 Department of Chemistry & Biochemistry, University of California San Diego, La Jolla, California, United States of America; Cardiff University, United Kingdom

## Abstract

Interleukin-1β (IL-1β) is the cytokine crucial to inflammatory and immune response. Two dominant routes are populated in the folding to native structure. These distinct routes are a result of the competition between early packing of the functional loops versus closure of the β-barrel to achieve efficient folding and have been observed both experimentally and computationally. Kinetic experiments on the WT protein established that the dominant route is characterized by early packing of geometrically frustrated functional loops. However, deletion of one of the functional loops, the β-bulge, switches the dominant route to an alternative, yet, as accessible, route, where the termini necessary for barrel closure form first. Here, we explore the effect of circular permutation of the WT sequence on the observed folding landscape with a combination of kinetic and thermodynamic experiments. Our experiments show that while the rate of formation of permutant protein is always slower than that observed for the WT sequence, the region of initial nucleation for all permutants is similar to that observed for the WT protein and occurs within a similar timescale. That is, even permutants with significant sequence rearrangement in which the functional-nucleus is placed at opposing ends of the polypeptide chain, fold by the dominant WT “functional loop-packing route”, despite the entropic cost of having to fold the N- and C- termini early. Taken together, our results indicate that the early packing of the functional loops dominates the folding landscape in active proteins, and, despite the entropic penalty of coalescing the termini early, these proteins will populate an entropically unfavorable route in order to conserve function. More generally, circular permutation can elucidate the influence of local energetic stabilization of functional regions within a protein, where topological complexity creates a mismatch between energetics and topology in active proteins.

## Introduction

Experimental studies using circularly permuted protein variants have given insight into the folding landscape and potential routes and mechanisms during folding for a variety of protein families [1]–[3]. While the order of secondary structural elements does not appear to be crucial in determining the folded protein structure, the arrangement of those elements may alter the preferred folding pathway [4] or increase the diversity of the available routes [5]. Recent experiments on S6 have shown that some proteins can also switch routes if an alternate folding nucleus becomes available when the native N- and C-termini are linked [6]. If a protein has one dominant folding route, it is possible that a change in the connectivity of the secondary structural elements can influence a change in folding rate [1], [7], [8]. In this regard, a protein may access less populated or entropically costly folding routes to overcome a cut in one of the folding nuclei, especially if that perturbation is in an area critical for development of the functionally relevant regions of the protein. Understanding the determinants of route selection becomes even more challenging as the function and folding landscapes necessarily overlap while nature optimizes folding for maximal function. Despite conservation of protein topology, we've shown that switching of the folding route occurs as a result of conversion of agonist to antagonist activity in IL-1β, via deletion of a functional loop [9]. This result suggests that a better understanding of the folding landscape may give novel insights into the production of designer proteins with partial agonist/antagonist activities.

**Figure 1:**
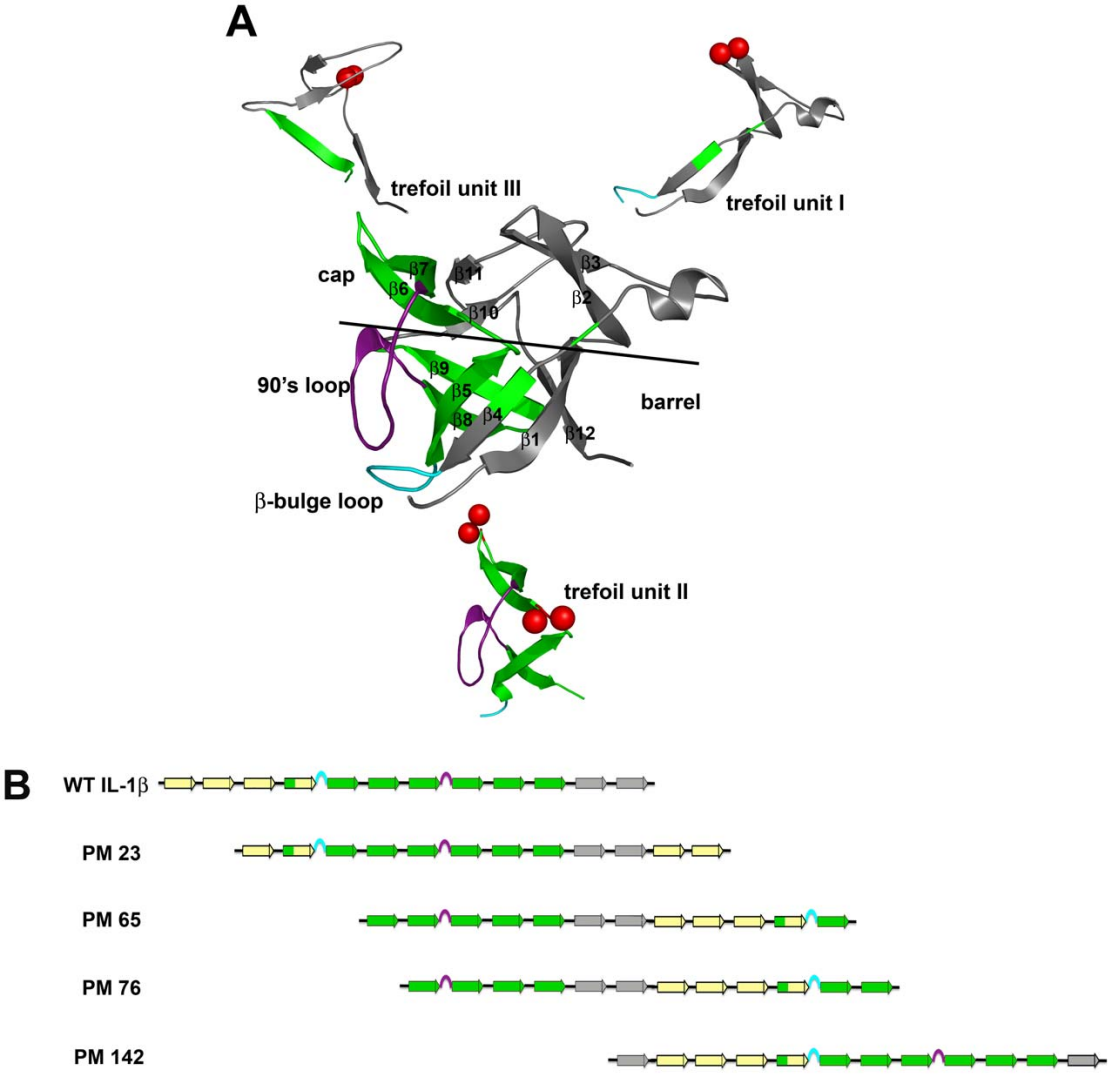
Schematic indicating the location of the permutations of IL-1β. (A) The molecule of IL-1β colored to indicate the strands involved in each trefoil unit. The black line delineates the cap and the barrel of the β-barrel protein. The subunits have been separated out to further indicate the spatial reference of the cut-sites that form the new N- and C- termini for each permutant, indicated by red spheres. PM23 (upper right) is located in a tight turn in the first trefoil subunit, between residues 22 and 23. PM65 (bottom) is also located in the central trefoil and is in a loop region between the cap and barrel strands, between residues 65 and 66. PM 76 (bottom) is located in the central trefoil subunit in a tight turn between two strands that form a β-sheet involved in the cap, between residues 75 and 76. Variants PM65 and PM76, both located in the second trefoil unit were designed in the middle of the intermediate structure that includes β-strands 5 through 10 (yellow strands). PM142 (upper left) is located in the long loop region of the third trefoil unit, between residues 141 and 142. The geometrically frustrated and functionally relevant loops are indicated in cyan (β-bulge) and purple (the 90′s loop), respectively. (B) Linear representations show how the N- and C-termini move relative to the two functional loops. Yellow β-strands indicate N-terminal β-strands, green β-strands highlight the region associated with the kinetic intermediate, and grey β-strands indicate C-terminal strands. The functional β-bulge and 90′s loops are highlighted in cyan and purple, respectively.

IL-1β is a three-fold pseudo-symmetric β-trefoil protein composed of three similar trefoil subunits (β-β-β-loop-β) (Fig. 1A). IL-1β has two identified binding sites, A and B. Both sites bind the IL-1 receptor, but only the B-site triggers a signal cascade [10], [11]. Both simulation [12] and experiment [13], [14] confirm that this robust protein folds via formation of a kinetic intermediate that is predominately composed of strands within the second (central) trefoil unit (β-strands in green, Fig. 1A). The β-strands within this region flank geometrically frustrated and functionally important loops, the β-bulge and the 90′s loop (Fig. 1A, cyan and purple, respectively), and aid in loop packing and orientation during folding. Both of these loops make up the B-site region [10], [11], which is present only in IL-1β, and is critical for signaling [10], [15]. Structure-based models indicate a rugged, heterogeneous folding landscape for IL-1β with multiple routes available [16], [17]. Different sites of initiation for folding characterize these various routes, i.e. barrel-closure first versus functional-loop packing, where the competition between the two routes creates another route, backtracking, and slows folding [16], [18]. Additionally, it has been suggested that functional loops that cause complexity and trapping, such as those found in IL-1β, can modulate folding routes and rates [19].

The dynamic energy landscape for IL-1β is malleable enough to allow for rearrangement of the peptide chain and provides an ideal system for the design of circular permutants that can help us further understand the contribution of functional regions during the folding process. Because it is established that there are multiple routes in the folding of IL-1β, with functional-loop packing having been initially confirmed experimentally [13], [14], followed by more recent demonstrations of both the backtracking [18] and barrel-closure routes [20], the question addressed here is: how do changes in connectivity effect the available routes in folding? In this article, four circular permutants of IL-1β: PM23, PM65, PM76 and PM142 that were amenable for study are highlighted. Linear representations show how the N- and C-termini move relative to the two functional loops in (Fig. 1B). PM 65 and PM76 have been cut within the WT folding intermediate region (second trefoil unit), and PM23 and PM142 have their cut-sites in the first and the third trefoil units, respectively, (Fig. 1B). Given that the WT protein folds to native first by coalescing around the second trefoil unit to pack the functional loops followed by packing the termini (functional loop packing), will the altered linear connectivity from chain rearrangement affect folding and/or the native fold? We use a combination of folding kinetics, hydrogen-deuterium exchange (HDX), and quench-flow pulse-labeling techniques to investigate the folding behavior and native ensemble of these IL-1β circular permutants.

As shown in this study, the permutant variants have the same global fold as WT IL-1β, confirming the proper packing and orientation of the functional loops. Interestingly, all permutant variants (both those with the cut-site within the kinetic intermediate and those with the cut-site elsewhere) exhibit similar rates of folding from the denatured/unfolded ensemble to the partially structured intermediate state. In addition, structural characterization of the intermediate for each of these constructs indicates that it is structurally similar to that observed for WT, with similar formed regions protected early in folding. However, the transition from the intermediate species to native is slower than WT for all permutants, most notably for those permutants cut within the WT intermediate region. In the S6 protein, circular permutation completely changes the folding transition state, suggesting changes in available routes for folding [6], [7]. In contrast, early formation and packing of the β-bulge and 90′s loop, both functional loops in IL-1β, appears to influence selection of the folding pathway in the permutation variants of IL-1β, so that the kinetic intermediate seen in the functionally relevant WT structure is maintained, as well as biological function [21]. These results highlight the unique interplay between folding and function, where route selection is mitigated by protection and packing of the functional loops, despite the entropic cost of the route selected.

## Results

### Circularly Permuted IL-1β Constructs have an Intact β-trefoil Superfold

**Figure 2:**
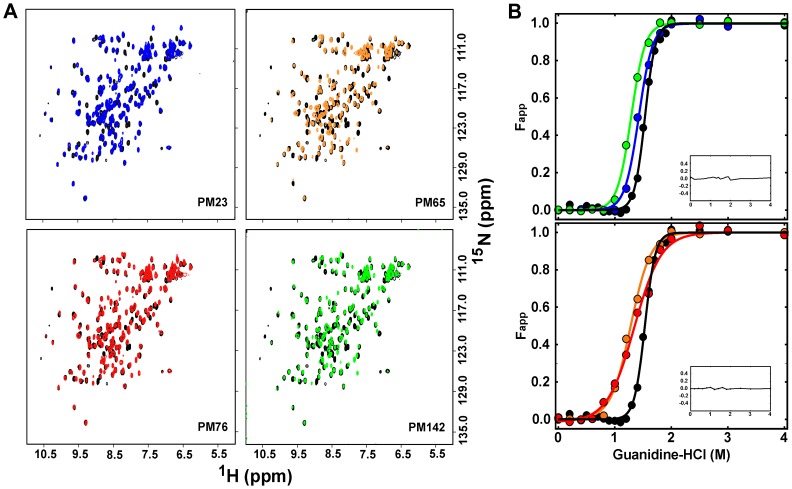
Plots of the (A) ^1^H-^15^N-HSQC spectra and (B) equilibrium titration of F_app_ as a function of denaturant concentration for WT-IL-1β and permutant proteins. (A) Representative spectra used to determine the effects of permutation on the global fold of the protein. Each permutant variant spectrum is overlaid with a spectrum of WT IL-1β (black). (Top, left) The spectrum of PM23 in overlaid blue. (Top, right) The spectrum of PM65 overlaid in orange. (Bottom, left) The spectrum of PM76 overlaid in red. (Bottom, right) The spectrum of PM142 overlaid in green. The overall pattern remains unaffected by circular permutation. (B) Following the same coloring scheme as above, equilibrium denaturation curves for WT IL-1β and circular permutant variants were plotted as F_app_ versus denaturation concentration. (Top) An overlay of WT-IL-1β plotted with PM23 and PM142. (Bottom). An overlay of WT IL-1β plotted with PM65 and PM76. Continuous lines represent the best-fit curves that are fit to a two-state model and are shown in the same color scheme. The residuals of the fit are shown as the inset.

Several permutants variants were attempted (Fig. S1A), including permutants within the functional loop regions of the protein. While all the permutant variants had a high predicted probability of forming [22], five (PM35, PM52, PM100, PM108 and PM129) were insoluble or prone to extensive aggregation (Fig. S1B) and were not characterized further. Permutant IL-1β proteins analyzed (PM23, PM65, PM76, and PM142) were expressed and purified using techniques developed on WT IL-1β. All optical and spectral data acquired (UV-Visible, fluorescence, CD, and homonuculear and heteronuculear data) confirm that initial assessment. In particular, NMR assignments show chemical shift patterns and dispersion quite similar to that observed for the WT [23], [24] (Fig. 2A).

### All IL-1β Permutant Variants are Destabilized Relative to the WT Protein

**Figure 3:**
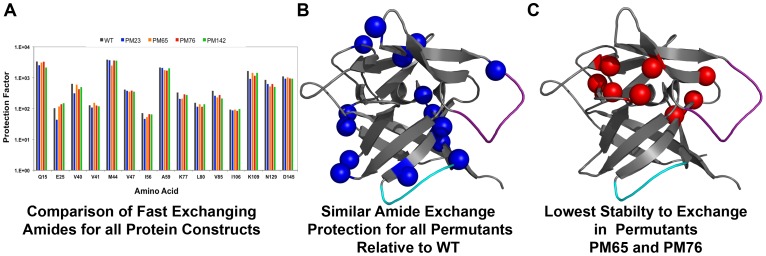
Summary of the native-state HDX indicates conservation of overall topology at the expense of individual backbone interactions. Molecular representations of the rates of HDX for the available backbone amide probes for all permutant variants compared to WT IL-1β. (A) Bar graph representing the calculated protection factors for the subset of backbone amides shown to exchange rapidly (fast exchanging) for WT and permutant variants of IL-1β. (B) Blue spheres represent the fast-exchanging residues that maintain similar protection for all permutant variants. These residues are located throughout the surface regions of the molecule. (C) Differences between the individual permutant variants mapped by red spheres. PM65 and PM76 both showed greater loss of protection in a handful of residues, located mostly within the hairpin cap. In particular, destabilization of the residues involved in β-strand packing of the hairpin cap lead to the greatest loss in cooperativity seen in all the variants (Fig. 2B).

Equilibrium chemical denaturation experiments were performed on all permutant variants to assess the effect of changes in chain connectivity on the thermodynamic properties relative to that observed for the WT protein (see Fig. 2B). Cut-sites in the first (PM23) and third (PM142) trefoil units of IL-1β exhibit destabilization to chemical denaturant relative to that observed for WT protein (Fig. 2B, upper), where the ΔΔG is −0.9±0.4 kcal/mol for PM23 and −1.5±0.5 kcal/mol for PM142, respectively (Table 1). Cut-sites within the second trefoil unit (PM65, PM76) show even more destabilization relative to the WT protein (Fig. 2B, lower), where the ΔΔG is −3.4±0.4 kcal/mol for PM65 and −3.7±0.2 kcal/mol for PM76, respectively (Table 1). In addition, there are changes in the cooperativity (*m*-value) of the folding transition relative to WT for all the permutants, with the second trefoil unit permutants, PM65 and PM76, showing the greatest differences, 3.4 kcal/mol*M and 2.9kcal/mol*M, respectively, compared to WT, 6.2 kcal/mol*M (Table 1). Comparison of the circular dichroism and fluorescence detected equilibrium transitions have shown no evidence for the population of equilibrium intermediate species [25] (Figure S2). This suggests that the decreased cooperativity of the unfolding transitions for PM65 and PM76 may be a result of changes in the native state dynamics in low levels of denaturant such that the barrel core is more solvent accessible, as seen for a handful of residues in the WT protein as a function of low levels of denaturant [23]. We performed NMR assignment methods (Figure 2A) as well as native-state HDX (Figure 3, Table S1, Figure S3) on the WT and all permutant proteins to compare the native states.

**Table 1:** Thermodynamic Parameters for WT IL-1β and All Permutant Variants.

IL-1β	ΔG_N-U_ a,b(kcalmol^−1^)	ΔΔG_N-U_ a,b(kcalmol^−1^)	*m*-value_N-U_ a,b(kcalmol^−1^M^−1^)	C*_m_* c (M)
**WT**	7.7±0.2	0	6.2±0.3	1.5±0.1
**PM23**	6.8±0.4	−0.9±0.4	4.8±0.2	1.4±0.1
**PM65**	4.3±0.3	−3.4±0.4	3.4±0.5	1.3±0.2
**PM76**	4.0±0.1	−3.7±0.2	2.9±0.3	1.3±0.1
**PM142**	6.2±0.5	−1.5±0.5	4.8±0.4	1.4±0.2

Changes in folding parameters upon circular permutating IL-1β at various turns and loops. The equilibrium data were fit using MATLAB in order to obtain equilibrium parameters for folding, ΔG_N-U_. Changes in ΔG_N-U_ (ΔΔG_N-U_) were obtained using WT as a reference. *m*-value indicates changes in the accessible surface area upon folding and indicate cooperativity of folding.

a Equilibrium transition data were evaluated using a two-state folding model.

b Data were obtained by calculating the average wavelength and calculating the relative average wavelength in terms of F_app_ as a function of [Gnd-HCl].

c C*_m_* values were taken from dividing the ΔG_N-U_ by the *m*-value.

### Characterization of Protein Dynamics by Native-state Amide Solvent Exchange Indicates Conservation of Surface Dynamics and Destabilization of the Hairpin Cap

Using native-state HDX to detect the rate of solvent exchange of individual backbone amide hydrogen atoms, the HDX rates were determined for ∼70 of 153 amide protons in WT and ∼60 amide protons in each permutant variant by NMR. A representative time course for exchange for all permutant proteins, compared to WT is given in Figure S3A. Comparison of the decay of amide proton signal over time demonstrates both similarities and differences compared to WT (Fig. S3B). Following the same numbering as WT IL-1β, Table S1 is a summary of calculated protection factors for all permutant variants of IL-1β. Several residues maintain the same slow exchanging behavior as WT IL-1β and are mapped in blue in Figure S4A.

Remarkably, while the common observation that backbone amide protons of residues that have low levels of protection (fast exchanging) are the most destabilized towards HDX when the native-state is destabilized, the fast-exchanging amide protons for all permutant variants maintain equivalent exchange rates with respect to the WT protein, despite significant changes in thermodynamic stability of the native state. The fast-exchanging backbone amide protons that exhibit similar levels of protection are: Q15, E25, V40, V41, M44, V47, I56, A59, K77, L80, V85, I106, K109, N129, and D145, and are represented in the bar graph (Fig. 3A) and as blue spheres mapped onto the molecule (Fig. 3B). These residues are located in turns and loops at the surface of the molecule.

Residues with significant differences in protection factors compared to WT (but were similar for all permutant proteins) are depicted with the red spheres in Figure S4B. The differences in protection factors are a result of the disruption of the local environment, including cut-sites, subsequently altering the rate of HDX. In each case, the difference resulted in faster levels of exchange, compared to that observed for the WT protein. Interestingly, PM65 and PM76 show more significant changes in backbone amide protons that are located within the hairpin cap and at the interface of the cap and barrel. These residues are highlighted as red spheres in Figure 3C. These residues are: L60, L62, C71, Q116, I122, S123, and L134. Interestingly, surface protection has been maintained (Fig. 3A, Fig. S4A) in each permutant variant while the residues at the cap-barrel interface and the core have been destabilized (Fig. 3B, Fig. S4B). This result is consistent with the proposal that loosening of the cap from the barrel is a result of geometric stress related to closing the β-barrel structure as a result of the rearrangement. This increases the competition between pinning the hairpin cap on the β-barrel and maintaining functional loop orientation and leads to the change in cooperativity seen in equilibrium denaturation studies (see Fig. 2B).

**Figure 4:**
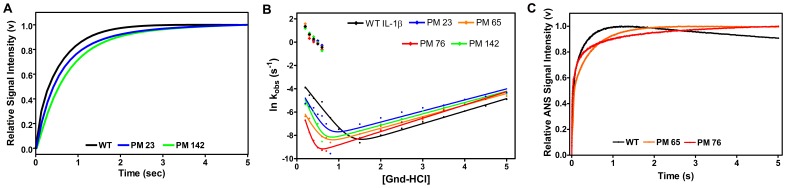
Representative traces of the folding kinetics and chevron plot of the relaxation rates indicating the effects of altered chain connectivity. (A) WT IL-1β is in black, PM23 in blue, and PM142 is in green. The traces are of the first 5 seconds of stopped-flow kinetic refolding jumps from 2.2 M Gdn-HCl to 0.4 M Gdn-HCl. Kinetic curves of WT and permutant proteins were fit to a three-exponential model. (B) Plot of the natural log of k_obs_ obtained by both stopped-flow and manual mixing refolding and unfolding experiments for all proteins variants as a function of final denaturant concentration. A comparison of the observed relaxation rates obtained by stopped-flow refolding experiments associated with the formation of the intermediate (upper left, diamonds) for WT IL-1β (black) indicating the similarities in observed rates of intermediate formation between PM23 (blue), PM65 (orange), PM76 (red), and PM142 (green). The data points (lower left and right, circles) and fits depict the rate of formation of the native protein. The differences in slope of the curves are a result of the cut site, where the greatest change in folding is seen in PM76 (red), indicating a slow transition to native. (C) ANS stopped-flow fluorescence-detected refolding of WT and permutant IL-1β. WT, in black, and permutant IL-1β protein (PM65 in orange, PM76 in red) were refolded from an unfolded state in the presence of ANS. The traces are representative of refolding jumps from 2.2 M to 0.3 M Gdn-HCl. The first 5 seconds is shown for clarity. The resulting curves confirm the similarity in the formation of the kinetic intermediate, but indicate a change in the release of the florophore.

### Fluorescence-detected Studies Indicate Alterations in the Folding Reactions

Time-dependent folding of WT and circular permutant variants of IL-1β were followed by fluorescence-detected stopped-flow and manual-mixing techniques. The kinetic behavior of folding as detected by these techniques for WT IL-1β exhibits a biphasic transition to the native state [25]–[27]. In the “fast phase”, monitored by stopped-flow, there is an increase in fluorescence on the hundreds of millisecond timescale [25]–[27]. Fitting the time-dependent intensity has been shown by quenched-flow data to be correlated with the population of a discrete, structured intermediate ensemble in WT IL-1β [13]. Figure 4A shows the first 5 seconds of a refolding reaction upon dilution from 2.2 M to 0.4 M Gdn-HCl for WT (in black), and permutants PM23 (in blue) and PM142 (in green), as monitored by stopped-flow fluorescence spectroscopy. These data are representative of the fast phase kinetic behavior seen for the refolding of all four permutant variants and in all cases, are independent of protein concentration, thus are not protein aggregation. The data indicate the biphasic behavior persists for all permutant proteins, consistent with that observed for the WT IL-1β and the population of a discrete structured intermediate ensemble (Fig. 4B, upper data points).

Fluorescence-detected manual mixing kinetics detects the formation of the native protein from the hyperfluorescent kinetic folding intermediate species in WT IL-1β [13]. The change in fluorescence intensity is on the tens of seconds to thousands of seconds′ timescale, depending on experimental conditions. In each case, the circular permutant proteins exhibit slower kinetics for the conversion of the intermediate to the native in the observed “slow phase” of folding of IL-1β. PM76, with its cut-site in the middle of the kinetic folding intermediate established for WT, is the slowest to reach native despite forming the intermediate in the same time frame as the WT protein (Fig. 4B, red circles, lower left). In addition, the rate of unfolding is increased for all permutant proteins, consistent with the destabilization of the native ensemble (Fig. 4B, colored circles, lower right).

Plots of the natural log of k_obs_ for refolding and unfolding of IL-1β permutant variants as a function of final denaturant concentration are presented in Figure 4B. The WT IL-1β data are represented in black. The fast phase of refolding for all permutant proteins studied is within error to that observed for WT protein (Fig. 4B, upper data points), while the slow phase of folding is significantly altered (Fig. 4B, low denaturant concentrations). Conversely, the unfolding of the WT protein is the slowest at all denaturant concentrations (Fig. 4B, high denaturant concentrations). Thus, transitions to the native, rather than intermediate state, is most affected by the rearrangement of the chain connectivity.

### 8-anilino-1-naphthalene Sulfonic Acid (ANS) Binding Studies are used to Probe the Formation and Dissipation of Partially Folded Species

Stopped-flow kinetic studies of the refolding of WT and the permutant IL-1β proteins were acquired in the presence of ANS (Fig. 4C). Intermediate formation has been followed using the fluorescence signal change that occurs as ANS first binds partially folded intermediate states and then dissociates as the native state ensemble is formed [28]–[30]. In IL-1β, ANS binds to hydrophobic clusters associated with the solvent exposed intermediate and the 90's functional loop region [15], [31], resulting in a large change in fluorescence intensity upon binding [31]. As Figure 4C demonstrates, the observed fast phase is linked to the formation of the kinetic folding intermediate and conservation of the packing of the functional loop region not only in WT, but in all permutant variants as well. In addition, similar kinetic rate values are observed as those determined in the absence of ANS (Fig. 4A). The release of ANS for some of the permutants (PM65 and PM76) is delayed compared to WT. We attribute this delayed release to a longer-lived intermediate species and increased backtracking [18].

### Variation in Linker size and Composition does not Affect Thermodynamic Stability, Cooperativity, or the Observed Rates of Folding

**Figure 5:**
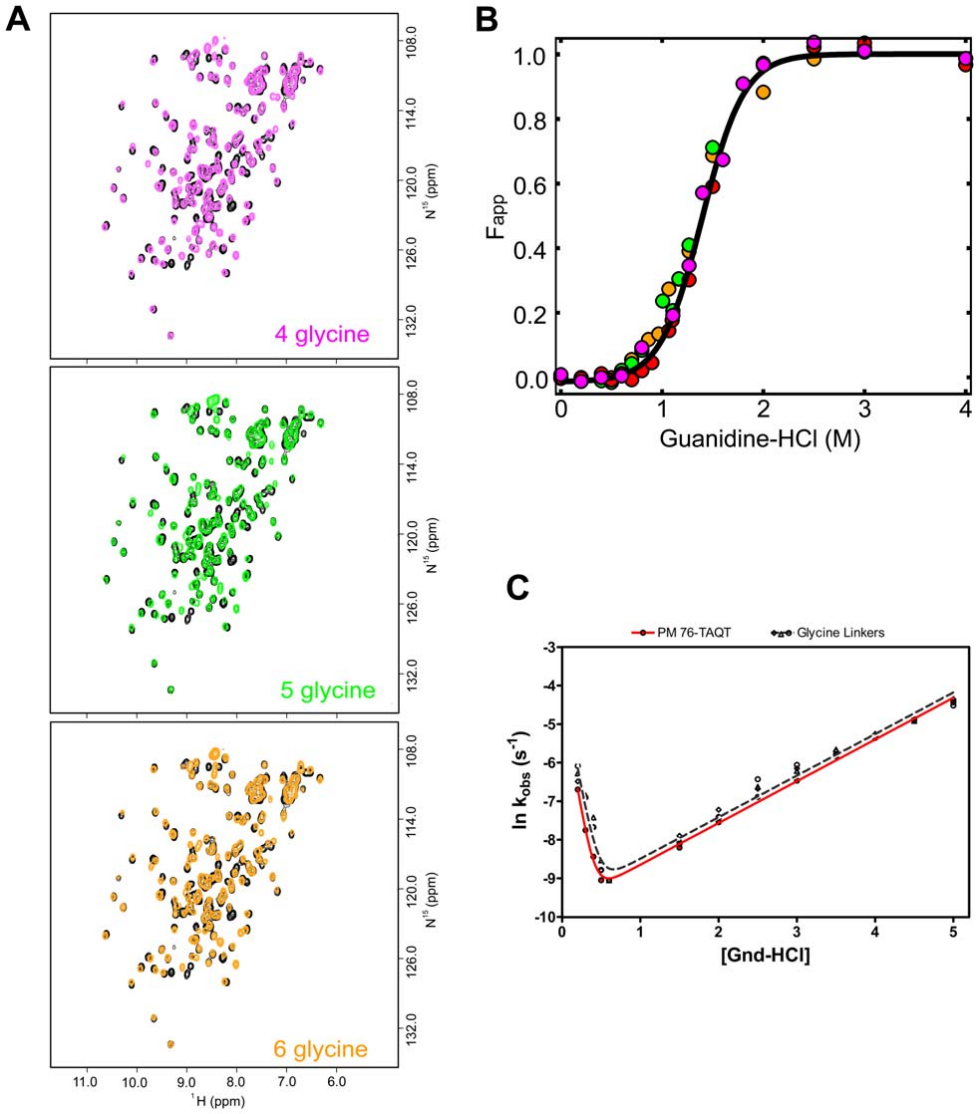
Plots of the (A) ^1^H-^15^N-HSQC spectra, (B) equilibrium titration and (C) chevron plot showing similar tertiary structure and no variation in equilibrium stability, cooperativity, or folding kinetics due to linker size or composition. (A) These representative spectra were acquired to determine the effects of linker length and composition on the global fold of the protein. Each linker variant spectrum is overlaid with the spectrum of WT IL-1β (black). (Top) The spectrum of the linker variant of PM76 with 4-glycine (4G) replacing TAQT overlaid in magenta. (Middle) The spectrum of the 5-glycine linker variant (5G) overlaid in green. (Bottom) The spectrum of the 6-glycine linker variant (6G) overlaid in orange. The overall pattern remains unaffected by the linker variations. (B) An overlay of linker variants equilibrium denaturation curves PM76 with the TAQT linker (red), 4G (magenta), 5G (green) and 6G (orange) plotted as F_app_ versus denaturation concentration. The continuous line representing the best-fit curve is fit to a two-state model is shown in black. (C) A chevron plot of the relaxation rates obtained by both stopped-flow and manual mixing refolding and unfolding experiments indicating the similarities in linker variants. The observed rates of PM76 with the biologically active TAQT linker (black data points) and 4G, 5G, and 6G (open data points) linker variants are plotted together.

We systematically altered the linker size and composition between the N- and C- termini in order to determine whether the observed thermodynamic and kinetic effects upon circular permutations could be partially the result of the presence of the biologically active linker, TAQT [21]. We chose to assess the role of the linker size and composition on the folding kinetics and thermodynamic stability of PM76 as this protein shows the biggest changes relative to WT (based on ΔΔG, m-value, κ_1_ and κ_2_) (see Fig. 2B, Table 1, and Fig. 4B). The HSQC spectra of WT IL-1β (black), and the three PM76 glycine linker variants (GGGG), (GGGGG), and (GGGGGG) are shown (Fig. 5A). All spectra are similar and indicate the composition of the linker does not alter the overall integrity of the native fold. In addition, equilibrium denaturation studies (Fig. 5B) and kinetic experiments (Fig. 5C) are within error of that observed for the original PM76 permutation. Taken together, these data indicate the size or composition of the linker does not alter the observed equilibrium or kinetic folding parameters for the permutant variants of IL-1β used in this study and that conclusions drawn can be attributed to the effects of changing the connectivity of the chain.

### HDX Pulse Labeling Indicates Conserved Nucleation Site for Intermediate Structures in WT and PM IL-1βs

**Figure 6:**
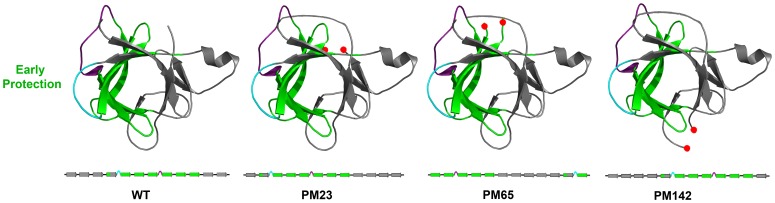
Summary plot of the results from the HDX pulse labeling experiments showing similar intermediate structure for WT and permutant proteins PM23, PM65, and PM142. Molecular and linear representations of IL-1β and permutants indicating β-strands involved in early folding events for all protein variants. The strands of the proteins protected early in the refolding (28 msec) reaction are colored in green for clarity. Purple and cyan highlight the functional loops, the 90′s loop and β-bulge, respectively. Red spheres represent the new termini. For all proteins, the β-strands within the functional route shows similar early protection in refolding. PM76 is not shown, as the protein variant was not amenable to these experimental conditions (see material and methods).

Using similar experimental conditions to those published recently [20] we performed HDX quench-flow pulse-labeling, monitored by NMR, to characterize the intermediate that is formed for each construct. The WT-IL-1β intermediate displays early backbone amide protection in the following residues: V40, F42, S43, V58, L60, L67, Y68, L69, S70, C71, V72, T79, Q81, E83, V85, V100, F101, N102, K103, I104, L110, E111, E113, S114, A115, Q116, F117, W120, Y121, I122, S123, which are predominately in β-strands 5–10 (Fig. 6, left). Each of the permutant constructs demonstrates similar early protection, regardless of the location of the cut-site (Table S2, Fig. 6, right). Differences appearing in the late forming strands are attributed to β-strands that participate in the backtracking event [18], where partial unfolding of the β-strands leads to loss of amide protection. Interestingly, the similarity in the kinetic intermediate β-strands further demonstrates the malleability of the landscape and of route selection. For example, in the case of the centrally cut permutants (PM65), the protein must bring what are now the terminal strands together in order to generate the same functional intermediate that is observed for the WT protein. Hence, early protection of the turns and strands encompassing the functional loops dominates the folding landscape for these signaling active permutant proteins.

## Discussion

### Structural Overview of the β-trefoil Fold of IL-1β

The β-trefoil fold is composed of 12 β-strands, 6 of which form a 6-stranded barrel (β-strands 1, 4, 5, 8, 9, 12) for the hairpin cap (β-strands 2, 3, 6, 7, 10, 11; Fig. 1A). Each of the hairpin cap β-strands is flanked by two barrel β-strands to form each of the three pseudo-symmetric (β-β-β-loop-β) subunits. Linear representations of WT and permutant proteins illustrate the frame shift of chain connectivity (Fig. 1B). In IL-1β, trefoil unit I (strands 1–3; loop with a 3_10_ helix; strand 4, Fig. 1A) contains binding site-A [10], [11] and the cut site of PM23 (between residues 23 and 24, Fig. 1A: upper right). The cut site of PM65 (between residues 65 and 66, Fig. 1A: bottom) lies at the edge of the experimentally determined kinetic folding intermediate region (strands 5–10, Fig. 1A: yellow strands) [13], [32] whereas PM76, (between residues 76 and 77, Fig. 1A, bottom), lies within the folding intermediate. The 90′s loop (between strands 7 and 8, Fig. 1A, purple) contributes to the geometric frustration and barrier to efficient folding as a result of packing with the β-bulge to form the trigger switch for the B-binding site [14]–[16]. Finally, the cut site PM142 (between residue 142 and 143, Fig. 1A: upper left) is within trefoil unit III (strands 9–11; loop; strand 12, Fig. 1A) at the leading edge of strand 12.

### Efficient Folding of Diverse Circular Permutants of IL-1β

Frame shifting the chain connectivity does not alter the overall fold of the permutant proteins relative to WT (Fig. 2A). However, it does result in decreased thermodynamic stability of the permutant proteins, with PM65 and PM76 showing the largest changes in native state stability (Fig. 2B, Table 1). Permutants that are cut within the WT kinetic folding intermediate region (PM65 and PM76), have the largest changes in cooperativity relative to permutants whose cut-sites are outside this region (PM23 and PM142; Table 1). However, all permutant proteins show differences in the rate of native-state solvent exchange for a subset of residues, indicating that the observed differences in stability result from an increase in fluctuations within the native state ensembles (Fig. 3C, Fig. S3B). For PM65 and PM76 (Fig. 3C) relative to that observed for the WT protein, this increased destabilization upon cutting within the kinetic intermediate may be further proof of the mismatch of energetics (the strength of the individual interactions) and topology (the complexity of the fold) created through circular permutation. Nonetheless, the rate of intermediate formation (Fig. 4) and the structure of the kinetic intermediate (Fig. 6) remain remarkably similar to that of WT protein, despite the entropic cost of bringing the ends together in the folding of PM65 and PM76. Simulations show that the three-fold pseudo-symmetry of IL-1β and its barrel structure allow multiple folding routes be populated to varying degrees during folding [16], [17]. These routes are distinct, where, in WT, formation, packing and orientation of the functional loops (function-packing route) dominates, followed by the barrel closure (coalescing of the termini β-strands first) and backtracking (allows for contact rearrangement to avoid folding traps) [16]. For IL-1β, the circular permutants generated compensate for the topological perturbation (i.e. the difficulty in folding) by switching between the multiple available native routes [16] to maintain the same folding/functional nucleus in the intermediate state. This is in contrast to circular permutants of 2-state proteins with one dominant folding route forming different folding nuclei [6]. Thus, altering the chain connectivity can increase the diversity of the available folding routes in more complex (non 2-state) yet symmetrical proteins [5], such as IL-1β, where permutant structures and folding landscape remain largely WT-like.

### Functional Influence on Route Switching

**Figure 7:**
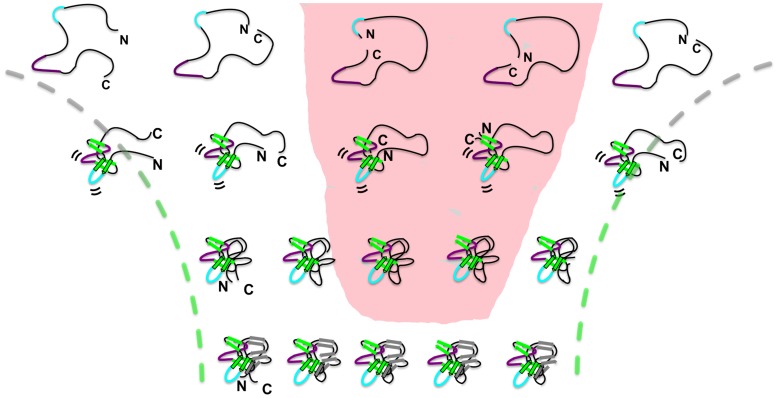
Multiple routes on the folding landscape of IL-1β. Schematic depiction of the progression of folding for WT IL-1β, (far left) PM23 (center left), PM65 (center), PM76 (center right), and PM142 (far right) proteins. The availability of multiple routes in IL-1β is exploited by the functional driving force allowing for efficient packing of the functional loops (and the maintenance of function) at the expense of efficient folding, where even entropically less-favored routes (pink shaded area) are selected to ensure proper functional orientation. In the formation of the intermediate in WT, the central trefoil unit collapses bringing the functional loops (in purple and cyan) in the proper conformation and orientation. The intermediate is the same for all the permutants but the route to the intermediate from the unfolded state varies. In the case of the centrally cut permutants (PM65 and PM76), the proteins still fold, albeit through an entropically less favorable route, as the termini must coalesce first to generate the same functional intermediate. The energetic payoff of forming a functional intermediate outweighs the entropic cost of bringing the termini together.

Experimental results demonstrated that switching between agonist/antagonist activities influences the selection of the folding routes for IL-1β [20]. In the present study of IL-1β permutant variants, all of which are biologically active receptor agonists [21], population of entropically less favorable routes to maintain the proper orientation of the functional signaling loops (Fig. 7) highlight the interplay between folding and function. It appears that the functional requirement (proper packing of the geometrically frustrated loops) sufficiently stabilizes the active region(s) in such a way that geometric changes imposed by permutations are not suitable enough to change the transition nucleus. Strikingly, the similarity in the amino acid composition of the kinetic intermediate for all the permutants is in contrast to previous reports where circular permutation resulted in changes in the folding nucleus [33] and appeared to fold by proximal interactions [5], [34]. IL-1β permutants appear to compensate for rearrangement by accessing less populated routes within the folding landscape in order to retain proper loop orientation and native structure (Fig. 7). For WT IL-1β (far left), the central trefoil unit collapses to form the initial folding nucleus. For permutants with cuts in the central trefoil unit, PM65 and PM76 (middle two in the shaded pink area), the terminal ends must be brought together to make the same functional nucleus. Despite the entropic cost of packing the N- and C- termini in the kinetic intermediate, and despite the decrease in global and local stability in the native ensemble, PM65 and PM76 use the diversity in available folding routes on the WT landscape to ensure correct functional loop positioning, overall native-like structure, and thus, protecting/maintaining biological function.

Importantly, the observed changes in kinetics for the formation and decay of this functional subunit (intermediate) are not imposed by the introduction of the artificial TAQT linker or by changes in the entropic penalty associated with the length of the linker (GGGG, GGGGG, GGGGGG) (Fig. 5). However, once the individual β-strands coalesce to form the native functional subunit (kinetic intermediate), and despite the entropic cost of the initial folding moment via the barrel-closure route, the interplay of multiple accessible routes, and the functional influence, is evident. As a result of changes in the long range contacts, an increase in backtracking [18], another accessible route necessary for the rearrangement of late forming regions to ensure proper folding, slows down the search for the native structure (Fig. 4B). This demonstrates the influential role of function over folding. A similar but more global interplay has the opposite effect in S6 [6].

Although WT IL-1β has been experimentally observed to fold through one dominate route that appears to be dictated by function [20], IL-1β can fold through a variety of routes in the folding/functional landscape. Such diversity of options in folding pathways gives flexibility to the IL-1β protein to adapt folding to the functional requirements and the geometric frustration of the molecule, and in this study, allows folding to adapt to a change in connectivity. Although a particular primary sequence usually has a dominant folding route, the alternative routes are just a few k_B_T's less stable, and therefore still accessible. Circular permutation of a symmetrical and complex protein, as we've demonstrated here with IL-1β, is a direct probe to explore how minor mismatches between entropy and local stabilization energy can be used to dissect the selection of a desired folding route and how it relates to function. The functional folding route for IL-1β underscores the folding-function interplay where early protection of the signaling nucleus encompassing the long-90′s loop and the β-bulge dominates the folding landscape, even when split between the two terminal positions. The IL-1β folding landscape has developed into multiple accessible routes that can be easily navigated to compensate for the difficulties associated with the formation of functionally constrained regions within the symmetric scaffold.

## Materials and Methods

### Construction of Permutant Proteins

A genetic engineering approach was adapted to create a DNA sequence composed of two human IL-1β cDNAs linked by a sequence of residues, TAQT, that were shown to have similar biological signaling behavior as WT IL-1β [21]. To construct the various permutant proteins of IL-1β, we used the four-primer PCR method [21] with some modifications. The tandem-gene permits construction of a diverse variety of IL-1β permutant proteins with the linker connecting the original N- and C- termini of IL-1β and then breaking the polypeptide chain between residues *i* and *i*+1, so that *i* and *i*+1 becomes the new N- and C- termini. Primers for construction of the template consisted of two IL-1β genes in tandem where two sets of primers yielded two IL-1β genes, connected by a linker region. The PCR conditions used for the construction of the IL-1β permutant proteins were similar to previously published [21]. To construct a permutant variant with varying linker length, we followed the same tandem-gene method, making modifications to two primers used to form the double-gene, incorporating the various linker lengths within the double gene.

### IL-1β Permutant Expression and Protein Purification

IL-1β permutations were expressed and purified according to methods previously published with some modifications [27]. The pellet of the insoluble fraction was dissolved in minimum amounts of 5.4 M guanidine isothiocynate, 25 mM Tris-HCl, 1 mM EDTA and 1 mM DTT at pH 7.5. It stood stirring for one hour at room temperature. The protein was refolded by adding 9 volumes of 50 mM KH_2_PO_4_ (pH 9.0), 1 mM EDTA, 1 mM DTT and 50 mM NaCl. It was allowed to stir at room temperature for 30 minutes. The solution was then centrifuged for 15 minutes at 4000×g (Sorvall RC-3). The supernatant was then dialyzed overnight into 4 liters of buffer A with 5 changes separated by a minimum of 3 hours. The dialysis was then centrifuged again. The resulting supernatant was then purified by ion exchange and size-exclusion chromatography like the soluble fraction as described previously. Uniform ^15^N labeling of IL-1β permutant protein was expressed according to methods previously published [23] and purified as above.

### NMR Spectroscopy

We performed ^1^H-^15^N HSQC experiments [35], [36] on the DMX 500 NMR Spectrometer (Bruker) for each of the permutant proteins constructed and WT IL-1β. All NMR experiments were performed similar to previous experiments [23]. Protein samples consisted of approximately 1 mM of protein in 100 mM deuterated ammonium acetate (Aldrich) and 10% D_2_O (Isotech) at pH 5.4. Experiments were run at 36°C. All experiments were processed using Felix 95.0 software (MSI, San Diego, CA).

Three-dimensional CBCA(CO)NH [37], [38] and HNCA [39] spectra were collected for permutated IL-1β protein samples that were approximately 0.5–0.7 mM in 100 mM sodium acetate-d_3_, pH 5.4, 10%/90% D_2_0/H_2_0 at 36°C. The CBCA(CO)NH spectra were collected with constant time in t_1_ and t_2_. For the ^1^H-^15^N-^13^C dimensions, a total of 64 complex t_1_ (^13^C), 32 complex t_2_ (^15^N), and 512 complex t_3_ (^1^H) points were collected. The spectral widths were 4024 Hz (32 ppm) (t_1_), 1520 Hz (30 ppm) (t_2_), and 3004.808 Hz (6 ppm)(t_3_). Points in the indirectly-detected dimensions were linear predicted and zero-filled. The HNCA spectra were collected as described [23] with constant time in t_1_. For the ^1^H-^15^N-^13^C dimensions, a total of 32 complex t_1_ (^15^N), 70 complex t_2_ (^13^C) and 512 complex t_3_ (^1^H) points were collected. The spectral widths were 1520.513 Hz (30 ppm) (t_1_), 4024.469 Hz (32 ppm) (t_2_) and 3004.808 Hz (6 ppm) (t_3_). Points in the indirectly detected dimensions were linear-predicted and zero-filled. Water suppression was achieved using a 3-9-19 train pulse sequence with gradients. When needed, broadband ^15^N decoupling during acquisition was accomplished using a WALTZ16 decoupling scheme. All experiments were processed using Felix 95.0 software (MSI, San Diego, CA).

### Stability Measurements

Equilibrium unfolding titrations were measured using average fluorescence wavelength [40], [41]. The single tryptophan residue in the IL-1β sequence was used to monitor the unfolding reaction. Trp 120 has been shown to be a useful probe of the global unfolding reaction [42] and was still a viable probe exhibiting relatively unchanged fluorescence despite the permutation. Protein samples were prepared at 0.2 mg/ml protein in a buffer solution (MES) containing varying concentrations of denaturant ranging from 0 to 4 M (Gdn-HCl). The protein solutions were prepared and fluorescence emission spectra were collected by methods previously published [27].

### Stopped-flow Fluorescence-detected Refolding

Refolding times faster than 10 seconds were measured using stopped-flow methods. Stopped-flow fluorescence experiments were carried out as previously described [27]. Typical refolding spectra were averaged over a minimum of 7 runs and a maximum of 14.

### Manual-mixing Fluorescence-detected Refolding/Unfolding

Refolding times greater than 10 seconds were measured using manual-mixing methods. Manual-mixing kinetics were carried out as previously described [27] on the Flouromax-2 spectroflourimeter (Spex) with the excitation wavelength set at 293 nm and the emission intensity monitored at 343 nm.

### ANS Stopped-flow Fluorescence-detected Refolding

Protein refolding prior to 20 seconds was monitored using stopped-flow methods. Stopped-flow fluorescence studies were performed with an Applied Photophysics Pi-Star SX.17MV instrument (Applied Photophysics, London) with a path length of 0.1 cm. The experiments were carried out similarly to as previously described [26].

### Amide H^N^ Solvent Exchange of IL-1β

Solvent amide NH exchange was measured as described previously [23] by following the change in intensity/volume of cross-peaks in the ^1^H-^15^N-HSQC spectra as a function of time. Following elution of the isotopically labeled protein from a spin column equilibrated in deuterated buffer [23], the first HSQC experiment for each HDX experiment was acquired uniformly at 26 minutes. A total of 80 HSQC spectra were collected in series, with 12 minutes for each spectrum at 36°C. The amide proton signal decay was plotted as a function of time for each individual amide resonance. A total of 67 residues were used for comparison between WT and permutant IL-1β variants. The change in intensity/volume of individual amino acid cross-peaks over time were compared to WT IL-1β amide exchange results that were collected and compared to results previously published [24] [14].

Solvent amide NH exchange NMR spectra data following the decay in intensity/volume of amino acid cross-peaks over time for each permutant were compared to WT IL-1β and fit to a single-exponential function as previously described [23]. The determined rates for the permutations were compared with those obtained for WT protein and protection factors for various amide protons in the proteins were estimated using the equation previously published [23]. All the data analyses were conducted with Matlab and/or GraphPad software.

### HDX Quench-flow Pulse Labeling

HDX quench-flow pulse labeling experiments were performed similar to previous experiments [13], [14] with minor modifications. HDX pulse labeling samples were prepared similar to previously described [13] with some modifications. Protein samples were unfolded overnight in 3 M Gdn-HCl, 50 mM ammonium acetate, pH5.0 at a final protein concentration of ∼2 mg/ml. For the quench-flow refolding experiments, the unfolded protein samples were allowed to refold for 28 and 157 msec, respectively, in 50 mM ammonium acetate (0.3 MGdn-HCl final, D_2_O buffer) and pulsed with 200 mM glycine-NaOH (1∶2 dilution, pH10.1, D_2_O buffer) for 22 msec. The exchange reaction was quenched by dilution into 0.5 M ammonium acetate (1∶2 dilution, pH4.7, D_2_O buffer). The proteins were left to fully refold (∼1 hour for the slowest refold, PM76) prior to being concentrated down to ∼600 ul. Control samples were prepared following the above protocol with native protein in order to determine the number of exchangeable probes available. In the case of PM76, the rate of refolding at the denaturant dilution used at the pulse step is slow enough that all probes are washed out resulting in no protected backbone amide signal.

### Data Analyses

Fitting of the fluorescence detected equilibrium unfolding data to a two-state model as described previously, [27] allows determination of the free energy of stabilization, ΔG. Manual-mixing and stopped-flow fluorescence kinetic data were fit as previously described [27]. Global analysis of WT and permutant IL-1β protein refolding data consistently fit best to three exponential processes. Analysis of the unfolding data consistently fit best to a single exponential process. All NMR experiments were processed using NMRPipe [43]. The resulting processed spectra were analyzed using Sparky [44]. For HDX pulse labeling, resonance peaks were selected and peak volumes integrated for each available amide probe. Peak volumes were normalized to the final protein concentration and compared. Molecular representations were prepared with MacPyMol [45] using PDB 9ILB.

## Supporting Information

Figure S1 **Overlay of the equilibrium titration curves monitored by both the fluorescence and CD spectroscopies as a function of denaturation concentration for PM76.** A comparison overlay of the CD (open circles) and fluorescence (solid red circles) as a function of denaturant. The curves are super-imposable and are consistent with a two-state model of equilibrium unfolding.
(TIF)

Figure S2 **A series of ^1^H-^15^N-HSQC spectra demonstrating the time course of HDX monitored by NMR for IL-1β and permutant variants and a comparison of H^N^ solvent exchange rates between WT IL-1β and permutant proteins.** (A) Each permutant (PM23 blue, PM65 orange, PM76 red, PM142 green) is overlaid together with WT (in black). The ^1^H-^15^N HSQC spectra indicate the change in peak intensity over time for all proteins and the similarities in the fingerprint pattern for each protein variant at the completion of the experiment. (B) Representative comparisons of the change in amide proton signals as a function of time after introduction into deuterated buffer for WT IL-1β (•), PM23 (•), PM65 (•), PM76 (•), and PM142 (•). The upper trace (residue I106) displays amide protons that are despite being fast exchanging in the WT protein, they maintain equivalent protection factors even in the most destabilized permutant proteins. The lower trace (residue I19) is representative of those observed that are less protected from exchange in the permutated proteins than the observed slow rate of exchange seen in WT.
(TIF)

Figure S3 **Summary of the observed native HDX results for WT and permutant proteins mapped to the structure of IL-1β indicating the regions that are (A) unperturbed and (B) decreased in stability to HDX as a function of permutation.**
(TIF)

Figure S4 **Summary of the observed native HDX results for WT and permutant proteins mapped to the structure of IL-1β indicating the regions that are (A) unperturbed and (B) decreased in stability to HDX as a function of permutation.**
(TIF)

Table S1 **Protection factors for WT IL-1β and permutant variants.**
(DOC)

Table S2 **Backbone amides identified in pulse labeling experiment and their secondary structure location.**
(DOC)
