# Supplementary figures and images for: Folding Circular Permutants of IL-1β: Route Selection Driven by Functional Frustration
Source: PLoS One. 2012 Jun 5;7(6):e38512. doi: 10.1371/journal.pone.0038512 (PMC3367917; doi:10.1371/journal.pone.0038512)

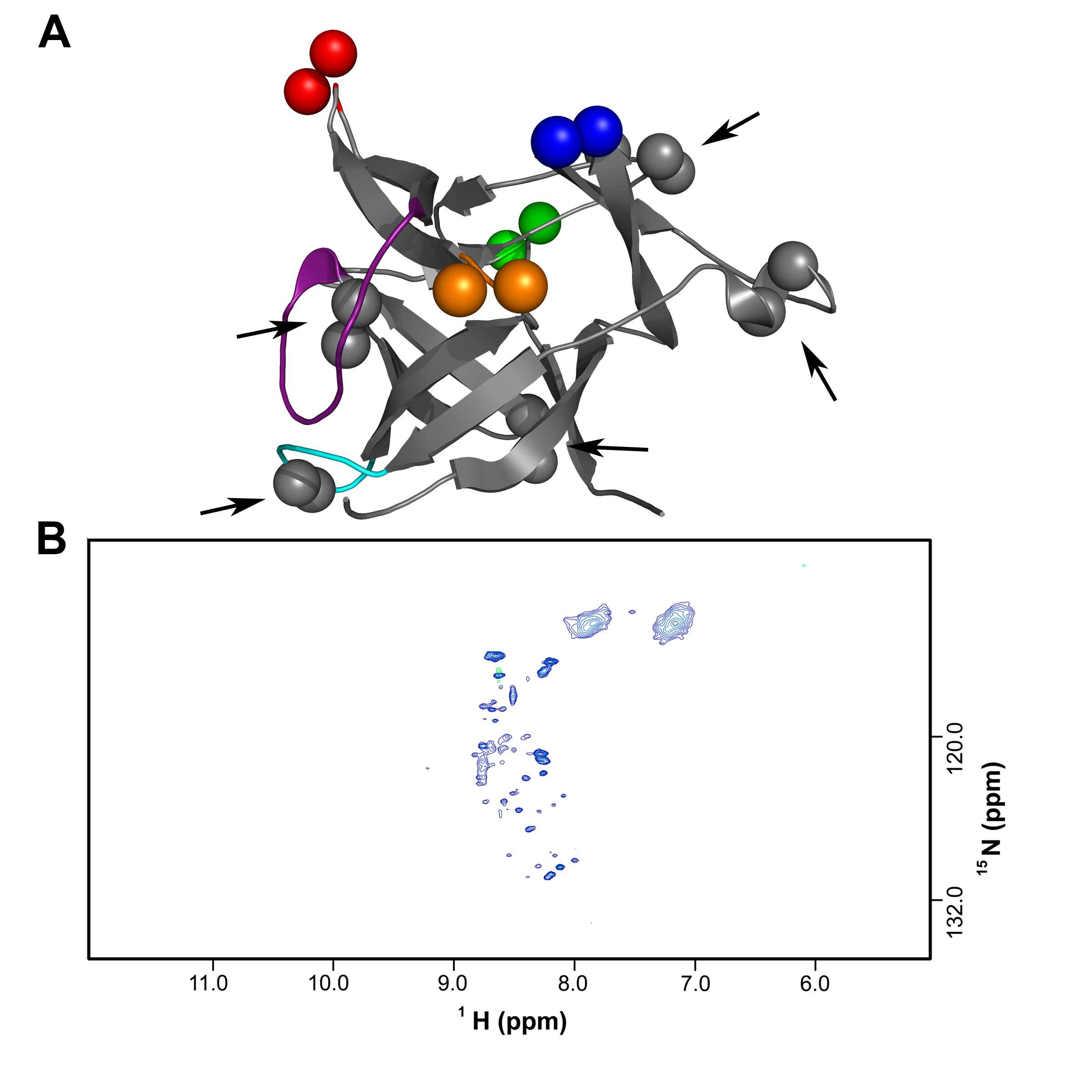

Supplement: Figure S1 — Overlay of the equilibrium titration curves monitored by both the fluorescence and CD spectroscopies as a function of denaturation concentration for PM76. A comparison overlay of the CD (open circles) and fluorescence (solid red circles) as a function of denaturant. The curves are super-imposable and are consistent with a two-state model of equilibrium unfolding. (TIF) [file pone.0038512.s001.tif]

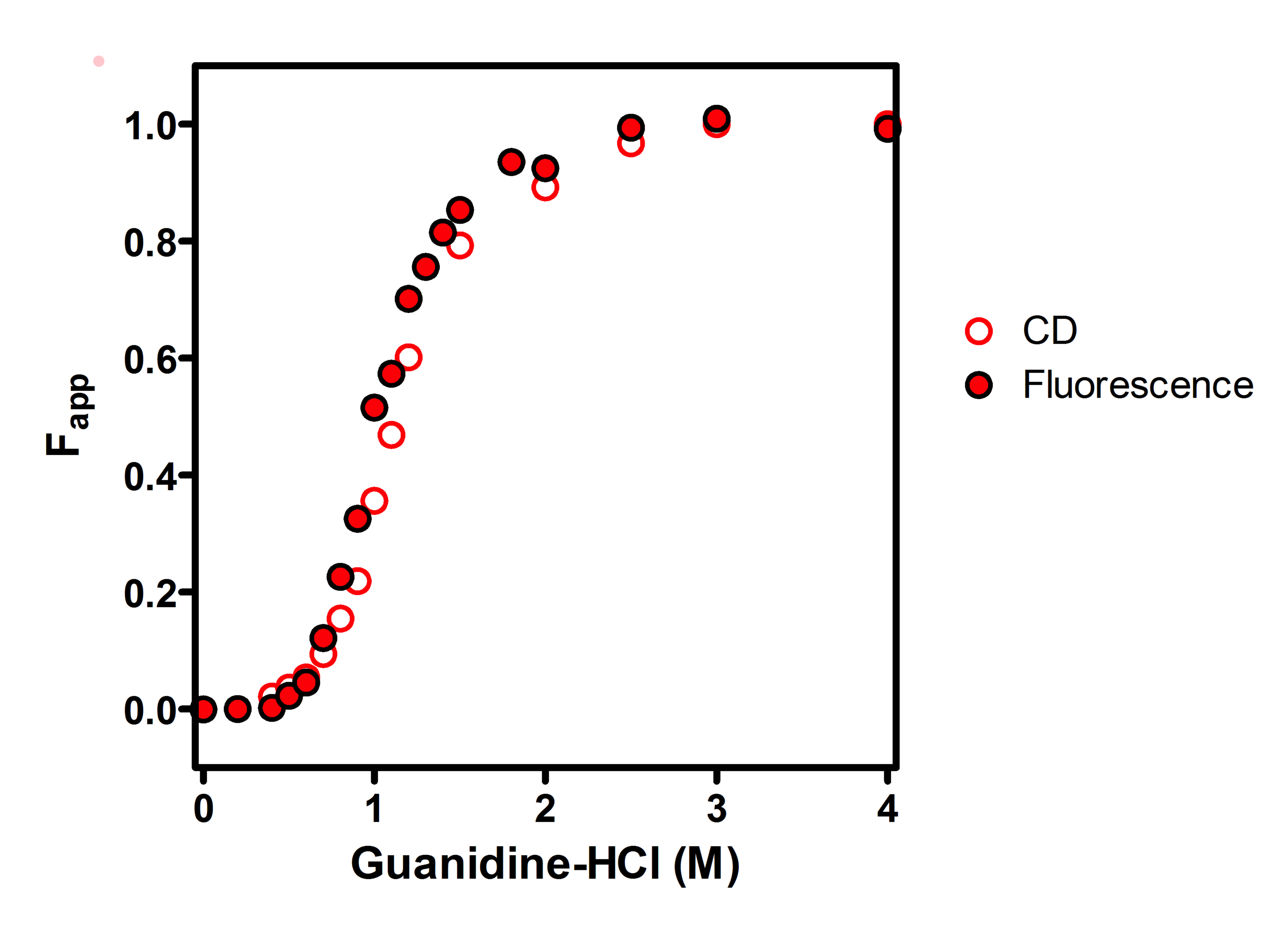

Supplement: Figure S2 — A series of 1H-15N-HSQC spectra demonstrating the time course of HDX monitored by NMR for IL-1β and permutant variants and a comparison of HN solvent exchange rates between WT IL-1β and permutant proteins. (A) Each permutant (PM23 blue, PM65 orange, PM76 red, PM142 green) is overlaid together with WT (in black). The 1H-15N HSQC spectra indicate the change in peak intensity over time for all proteins and the similarities in the fingerprint pattern for each protein variant at the completion of the experiment. (B) Representative comparisons of the change in amide proton signals as a function of time after introduction into deuterated buffer for WT IL-1β (•), PM23 (•), PM65 (•), PM76 (•), and PM142 (•). The upper trace (residue I106) displays amide protons that are despite being fast exchanging in the WT protein, they maintain equivalent protection factors even in the most destabilized permutant proteins. The lower trace (residue I19) is representative of those observed that are less protected from exchange in the permutated proteins than the observed slow rate of exchange seen in WT. (TIF) [file pone.0038512.s002.tif]

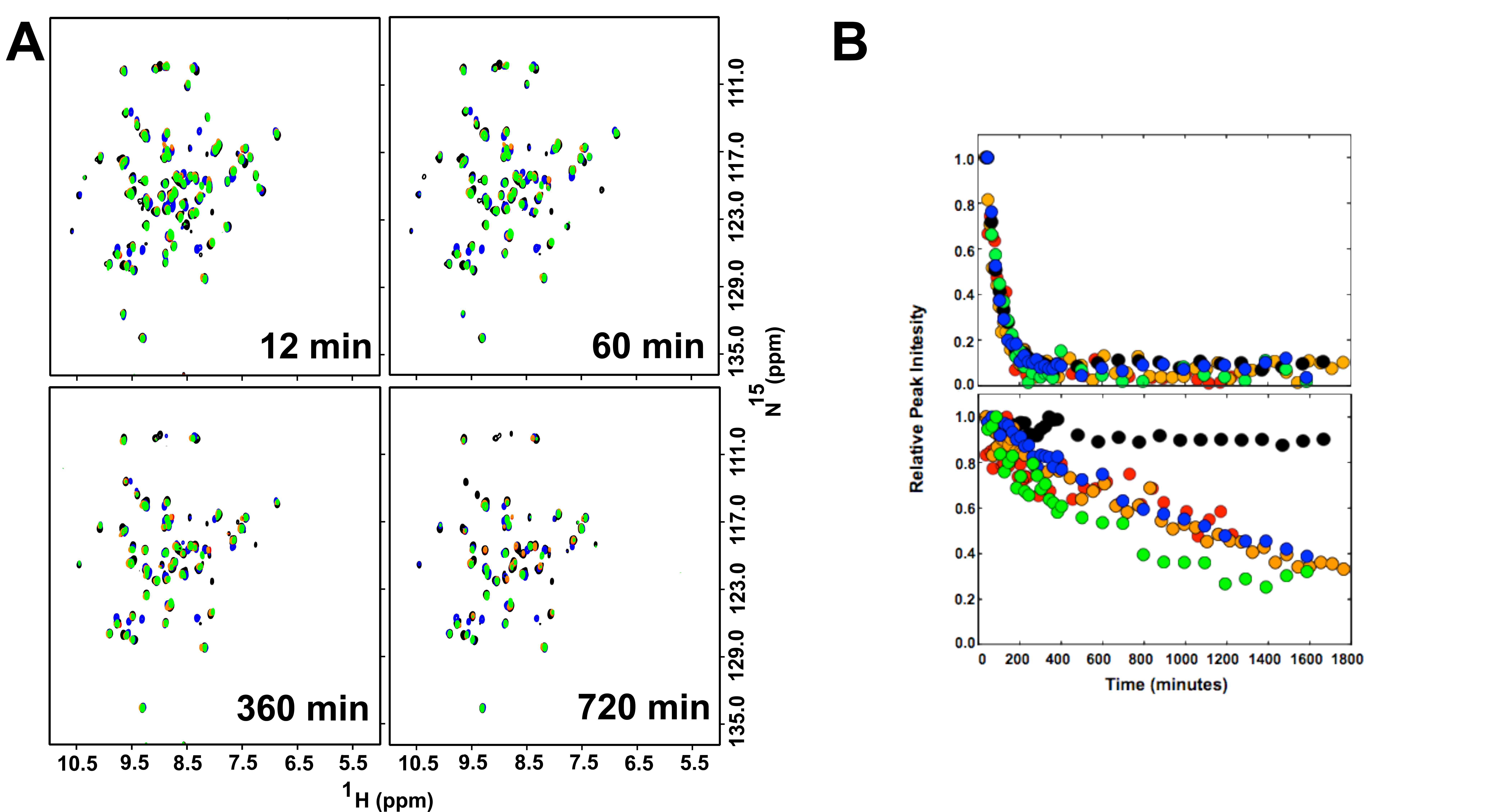

Supplement: Figure S3 — Summary of the observed native HDX results for WT and permutant proteins mapped to the structure of IL-1β indicating the regions that are (A) unperturbed and (B) decreased in stability to HDX as a function of permutation. (TIF) [file pone.0038512.s003.tif]

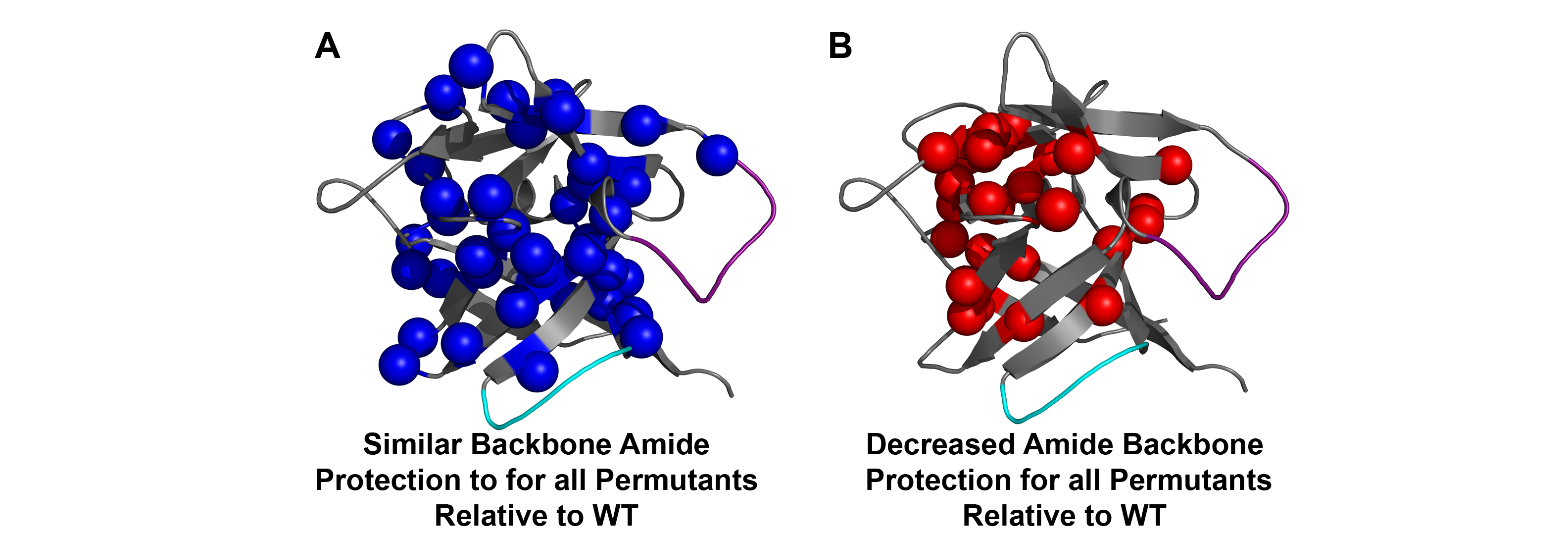

Supplement: Figure S4 — Summary of the observed native HDX results for WT and permutant proteins mapped to the structure of IL-1β indicating the regions that are (A) unperturbed and (B) decreased in stability to HDX as a function of permutation. (TIF) [file pone.0038512.s004.tif]
