# Supplementary material for: Folding Circular Permutants of IL-1β: Route Selection Driven by Functional Frustration
Source: PLoS One. 2012 Jun 5;7(6):e38512. doi: 10.1371/journal.pone.0038512 (PMC3367917; doi:10.1371/journal.pone.0038512)
Supplement: Table S1 — Protection factors for WT IL-1β and permutant variants. (DOC) [file pone.0038512.s005.doc]

| **SI Table 1: Protection factors for WT IL-1β and permutant variants** | | | | | | |
| --- | --- | --- | --- | --- | --- | --- |
| **Residue** | | **WT** | **PM23** | **PM65** | **PM76** | **PM142** |
| **8** | **C** | >1.0E+06 | 5.0E+04 | 9.5E+04 | 7.2E+04 | 2.3E+04 |
| **9** | **T** | 1.6E+04 | 1.2E+04 | 1.5E+04 | 1.1E+04 | 1.0E+04 |
| **10** | **L** | >1.0E+06 | 3.3E+03 | 5.0E+03 | 7.3E+03 | 8.7E+03 |
| **11** | **R** | >1.0E+06 | 8.3E+03 | 6.5E+03 | 3.2E+03 | 5.5E+03 |
| **12** | **D** | 3.8E+04 | 7.2E+03 | 1.3E+04 | 1.8E+04 | 5.3E+03 |
| **15** | **Q** | 3.4E+03 | 2.6E+03 | 3.1E+03 | 3.3E+03 | 2.1E+03 |
| **16** | **K** | 3.7E+04 | 8.8E+03 | 1.8E+04 | 1.2E+04 | 8.2E+03 |
| **17** | **S** | 5.4E+04 | 2.0E+03 | 4.0E+03 | 2.4E+03 | 2.9E+03 |
| **18** | **L** | 2.7E+04 | 3.2E+03 | 8.7E+03 | 2.8E+03 | 3.7E+03 |
| **19** | **V** | >1.0E+06 | 1.6E+03 | 2.0E+03 | 1.2E+03 | 7.5E+02 |
| **25** | **E** | 1.0E+02 | 4.4E+01 | 1.2E+02 | 1.4E+02 | 1.5E+02 |
| **27** | **K** | 1.8E+04 | 4.3E+03 | 4.4E+03 | 4.7E+03 | 5.4E+03 |
| **28** | **A** | >1.0E+06 | 9.6E+03 | 1.7E+04 | 9.8E+03 | 8.0E+03 |
| **29** | **L** | >1.0E+06 | 3.9E+03 | 5.7E+03 | 2.8E+03 | 1.9E+03 |
| **40** | **V** | 6.3E+02 | 3.1E+02 | 6.0E+02 | 4.3E+02 | 4.9E+02 |
| **41** | **V** | 1.3E+02 | 1.1E+02 | 1.5E+02 | 1.3E+02 | 1.2E+02 |
| **42** | **F** | 4.4E+04 | 3.3E+04 | 4.4E+04 | 3.3E+04 | 3.9E+04 |
| **43** | **S** | 1.7E+05 | 1.5E+05 | 1.6E+05 | 1.3E+05 | 1.7E+05 |
| **44** | **M** | 3.9E+03 | 3.7E+03 | 2.5E+03 | 3.6E+03 | 3.6E+03 |
| **45** | **S** | 1.4E+04 | 1.0E+04 | 1.1E+04 | 1.5E+04 | 1.2E+04 |
| **47** | **V** | 4.1E+02 | 3.8E+02 | 3.5E+02 | 3.8E+02 | 3.5E+02 |
| **56** | **I** | 7.1E+01 | 4.6E+01 | 5.4E+01 | 6.6E+01 | 6.4E+01 |
| **58** | **V** | >1.0E+06 | 3.3E+03 | 2.0E+03 | 1.3E+04 | 1.3E+03 |
| **59** | **A** | 2.1E+03 | 2.1E+03 | 1.7E+03 | 1.7E+03 | 2.0E+03 |
| **60** | **L** | >1.0E+06 | 7.2E+03 | 1.9E+03 | 1.8E+03 | 7.0E+03 |
| **61** | **G** | >1.0E+06 | 9.0E+03 | 1.7E+04 | 5.9E+04 | 6.5E+03 |
| **62** | **L** | >1.0E+06 | 6.8E+02 | 2.2E+01 | 4.1E+01 | 5.2E+02 |
| **67** | **L** | 4.2E+03 | 4.3E+03 | 4.0E+03 | 4.6E+03 | 5.1E+03 |
| **68** | **Y** | 3.5E+04 | 2.9E+04 | 1.7E+04 | 3.5E+04 | 2.9E+04 |
| **69** | **L** | >1.0E+06 | 3.0E+04 | 1.6E+04 | 2.4E+04 | 2.6E+04 |
| **70** | **S** | 9.0E+04 | 7.9E+04 | 7.4E+04 | 6.5E+04 | 7.9E+04 |
| **71** | **C** | >1.0E+06 | 4.3E+04 | 6.1E+03 | 4.1E+03 | 3.7E+04 |
| **72** | **V** | 6.0E+04 | 3.5E+04 | 5.0E+04 | 6.0E+04 | 5.0E+04 |
| **77** | **K** | 3.3E+02 | 2.1E+02 | 2.1E+02 | 2.9E+02 | 2.7E+02 |
| **79** | **T** | 5.1E+04 | 2.3E+04 | 2.5E+04 | 2.1E+04 | 2.6E+04 |
| **80** | **L** | 1.5E+02 | 1.1E+02 | 1.4E+02 | 1.1E+02 | 1.4E+02 |
| **81** | **Q** | 7.3E+04 | 2.5E+04 | 3.7E+04 | 2.4E+04 | 6.1E+04 |
| **83** | **E** | 2.4E+04 | 1.7E+04 | 1.2E+04 | 1.6E+04 | 2.1E+04 |
| **85** | **V** | 3.8E+02 | 2.6E+02 | 2.3E+02 | 2.8E+02 | 2.1E+02 |
| **99** | **F** | 2.7E+04 | 1.6E+04 | 1.2E+04 | 1.8E+04 | 1.2E+04 |
| **100** | **V** | >1.0E+06 | 1.2E+05 | 1.0E+05 | 2.1E+05 | 2.0E+05 |
| **101** | **F** | >1.0E+06 | 3.4E+05 | 2.2E+05 | 3.7E+05 | 3.7E+05 |
| **102** | **N** | >1.0E+06 | >1.0E+06 | >1.0E+06 | >1.0E+06 | >1.0E+06 |
| **103** | **K** | >1.0E+06 | 3.4E+04 | 3.8E+04 | 5.3E+03 | 2.4E+04 |
| **104** | **I** | >1.0E+06 | >1.0E+06 | >1.0E+06 | >1.0E+06 | >1.0E+06 |
| **106** | **I** | 9.2E+01 | 8.7E+01 | 9.3E+01 | 8.5E+01 | 9.7E+01 |
| **109** | **K** | 1.6E+03 | 9.2E+02 | 1.4E+03 | 1.2E+03 | 1.4E+03 |
| **110** | **L** | 7.3E+04 | 2.8E+04 | 4.2E+03 | 6.1E+03 | 2.4E+04 |
| **111** | **E** | >1.0E+06 | 1.0E+04 | 4.7E+03 | 2.1E+04 | 6.6E+03 |
| **113** | **E** | >1.0E+06 | 4.0E+04 | 2.9E+04 | 2.4E+04 | 3.8E+04 |
| **114** | **S** | >1.0E+06 | >1.0E+06 | >1.0E+06 | >1.0E+06 | >1.0E+06 |
| **115** | **A** | 1.9E+06 | >1.0E+06 | >1.0E+06 | >1.0E+06 | >1.0E+06 |
| **116** | **Q** | 3.3E+04 | 2.9E+03 | 1.3E+02 | 1.3E+02 | 3.7E+03 |
| **117** | **F** | >1.0E+06 | >1.0E+06 | >1.0E+06 | >1.0E+06 | >1.0E+06 |
| **120** | **W** | 8.4E+04 | 8.1E+04 | 4.2E+04 | 4.4E+04 | 7.1E+04 |
| **121** | **Y** | 4.4E+04 | 4.2E+04 | 2.2E+04 | 4.4E+04 | 3.7E+04 |
| **122** | **I** | 2.2E+04 | 1.7E+03 | 4.2E+02 | 2.9E+02 | 1.6E+03 |
| **123** | **S** | 1.4E+05 | 1.4E+04 | 2.2E+03 | 1.4E+03 | 1.2E+04 |
| **124** | **T** | 1.7E+05 | 1.1E+04 | 1.0E+04 | 8.1E+03 | 5.8E+03 |
| **125** | **S** | 3.8E+05 | 2.4E+04 | 3.6E+04 | 2.0E+04 | 1.8E+04 |
| **129** | **N** | 8.5E+02 | 6.3E+02 | 5.2E+02 | 6.3E+02 | 5.1E+02 |
| **132** | **V** | 1.3E+04 | 1.4E+03 | 2.5E+03 | 1.1E+03 | 1.1E+03 |
| **133** | **F** | 4.4E+04 | 3.3E+03 | 1.5E+04 | 8.8E+02 | 3.2E+03 |
| **134** | **L** | 3.2E+04 | 5.1E+03 | 4.6E+02 | 1.0E+02 | 4.1E+03 |
| **135** | **G** | 1.2E+05 | 7.0E+03 | 7.4E+03 | 5.6E+03 | 5.7E+03 |
| **145** | **D** | 1.1E+03 | 9.4E+02 | 1.0E+03 | 9.5E+02 | 9.3E+02 |
| **146** | **F** | 1.6E+04 | 1.3E+04 | 1.4E+04 | 1.2E+04 | 1.0E+04 |
| **147** | **T** | 1.0E+05 | 8.0E+03 | 1.3E+04 | 8.6E+03 | 1.1E+04 |
