# Supplementary material for: Folding Circular Permutants of IL-1β: Route Selection Driven by Functional Frustration
Source: PLoS One. 2012 Jun 5;7(6):e38512. doi: 10.1371/journal.pone.0038512 (PMC3367917; doi:10.1371/journal.pone.0038512)
Supplement: Table S2 — Backbone amides identified in pulse labeling experiment and their secondary structure location. (DOC) [file pone.0038512.s006.doc]

| **SI Table 2: Backbone amides identified in pulse labeling experiment and their secondary structure location** | | | | |
| --- | --- | --- | --- | --- |
| **WT** | **PM23** | **PM65** | **PM142** | **Location** |
| V40 | V40 | V40 | V40 | β4 |
| F42 | F42 | F42 | F42 | β4 |
| S43 | S43 | S43 | S43 | β4 |
| V58 |  | V58 | V58 | β5 |
| L60 | L60 | L60 |  | β5 |
| L67 |  |  | L67 | β6 |
| Y68 | Y68 |  | Y68 | β6 |
| L69 | L69 | L69 | L69 | β6 |
| S70 | S70 | S70 | S70 | β6 |
| C71 | C71 | C71 | C71 | β6 |
| V72 | V72 |  | V72 | β6 |
| T79 | T79 | T79 | T79 | β7 |
| Q81 | Q81 | Q81 | Q81 | β7 |
| E83 |  | E83 | E83 | β7 |
| V85 | V85 |  | V85 | β7 |
| V100 | V100 |  | V100 | β8 |
| F101 | F101 | F101 | F101 | β8 |
| N102 | N102 | N102 | N102 | β8 |
| K103 | K103 | K103 | K103 | β8 |
| I104 | I104 | I104 | I104 | β8 |
| L110 |  | L110 | L110 | β9 |
| E111 | E111 | E111 | E111 | β9 |
| E113 | E113 |  | E113 | β9 |
| S114 | S114 | S114 | S114 | β9 |
| A115 | A115 | A115 | A115 | β9 |
| Q116 | Q116 |  | Q116 | β9 |
| F117 | F117 | F117 | F117 | β9 |
| W120 | W120 | W120 | W120 | β10 |
| Y121 | Y121 | Y121 | Y121 | β10 |
| I122 | I122 | I122 | I122 | β10 |
| S123 | S123 | S123 | S123 | β10 |
